# Optimal Design for Hetero-Associative Memory: Hippocampal CA1 Phase Response Curve and Spike-Timing-Dependent Plasticity

**DOI:** 10.1371/journal.pone.0077395

**Published:** 2013-10-24

**Authors:** Ryota Miyata, Keisuke Ota, Toru Aonishi

**Affiliations:** 1 Interdisciplinary Graduate School of Science and Engineering, Tokyo Institute of Technology, Kanagawa, Japan; 2 Research Fellow of the Japan Society for the Promotion of Science, Tokyo, Japan; 3 Brain Science Institute, RIKEN, Saitama, Japan; Tokai University, Japan

## Abstract

Recently reported experimental findings suggest that the hippocampal CA1 network stores spatio-temporal spike patterns and retrieves temporally reversed and spread-out patterns. In this paper, we explore the idea that the properties of the neural interactions and the synaptic plasticity rule in the CA1 network enable it to function as a hetero-associative memory recalling such reversed and spread-out spike patterns. In line with Lengyel’s speculation (Lengyel et al., 2005), we firstly derive optimally designed spike-timing-dependent plasticity (STDP) rules that are matched to neural interactions formalized in terms of phase response curves (PRCs) for performing the hetero-associative memory function. By maximizing object functions formulated in terms of mutual information for evaluating memory retrieval performance, we search for STDP window functions that are optimal for retrieval of normal and doubly spread-out patterns under the constraint that the PRCs are those of CA1 pyramidal neurons. The system, which can retrieve normal and doubly spread-out patterns, can also retrieve reversed patterns with the same quality. Finally, we demonstrate that purposely designed STDP window functions qualitatively conform to typical ones found in CA1 pyramidal neurons.

## Introduction

It has been reported that characteristic ensemble spiking patterns are consistently repeated in the hippocampal CA1 region during waking and sleep periods [1]–[9]. Louie and Wilson (2001) reported that in rats, spike patterns produced during rapid eye movement (REM) episodes are very similar to those observed while the animals are running [8]. There are cases in which the timescale of these reactivation patterns during REM episodes is on average twice as long as that of the running periods. Foster and Wilson (2006) reported that the spike patterns observed during running periods are reproduced in a temporally reversed order during rest periods [9]. These experiments suggest that the CA1 network stores spatio-temporal spike patterns and retrieves reversed and spread-out patterns.

These experimental results raise a big question as to whether the hippocampal network including the CA1 and CA3 regions has an optimal structure for storing and retrieving such spike patterns. Lengyel et al. (2005) [10] developed a normative theory for auto-associative memory networks that specifies optimal pairs of the synaptic plasticity rule for embedding memories and the form of neural interactions for auto-associative memory retrieval. Under the speculation that a phase response curve (PRC) is appropriate to formulate the neural interactions if memories are embedded by spike-timing-dependent plasticity (STDP), they derived pairs of STDP window functions and PRCs optimally functioning as an auto-associative memory. They showed that the features of the PRCs of hippocampal CA3 pyramidal neurons qualitatively conform to ones theoretically derived from typical STDP window functions. However, they asked the question only as it relates to restoring phase patterns to the original stored state through mutual recurrent interactions, not retrieval of reversed and spread-out patterns. Moreover, although the possible existence of STDP at recurrent synapses between CA3 pyramidal neurons has been suggested [11], [12], as far as we know, there are no reports on capturing the entire shape of the STDP window function.

**Figure 1:**
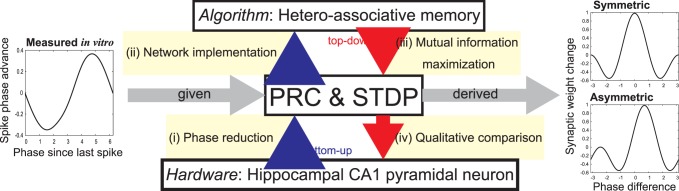
Outline of our approach. We derive pairs of PRCs and STDP window functions optimally recalling normal, reversed, and spread-out memory spike patterns.

Here, we focus on the CA1 network in which STDP has been reported [13]–[17]. We explore the idea that the properties of the neural interactions and the synaptic plasticity rule support the function of hetero-associative memory in which spike patterns are embedded in synapses and reversed and spread-out patterns are retrieved. In line with Lengyel’s speculation, we search for optimal pairs of STDP window functions and PRCs. Whereas Lengyel et al. used a top-down approach, treating the auto-associative memory retrieval as optimal probabilistic inference and inferring the retrieval dynamics that are normatively matched to the typical STDP window functions, we take a synthetic approach of optimal design for hetero-associative memory under the physical limitations of the neural implementation. Figure 1 illustrates our approach, consisting of bottom-up and top-down steps. In the bottom-up steps, under the assumption of regular firing and weak coupling, we firstly formulate a hetero-associative memory network recalling not only the normal spike patterns, but also the reversed and doubly spread-out patterns as a phase oscillator model consisting of an STDP window function and a PRC. This network model associates pre- and postsynaptic phase patterns. For example, when presented with a stored presynaptic phase pattern that is temporally reversed or spread out, the postsynaptic neurons can recall the associated phase pattern that is temporally reversed or spread out (for more detail, see Table 1). Secondly, we analytically derive the mutual information between a stored phase pattern and a network output, and use it to evaluate memory retrieval performance. In the top-down steps, by maximizing the objective function given by the mutual information, we search for a set of optimal STDP window functions under the constraint of PRCs recorded *in vitro* from hippocampal CA1 pyramidal neurons [18], [19].

**Table 1:** Outline of hetero-associative memory functions we studied.

	Presynaptic phase	Postsynaptic phase
Storage process	Memory key pattern	Memory output pattern
	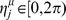	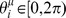
Retrieval process	Retrieval key pattern	Retrieval output pattern
	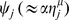	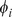 ( 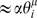 )

Phase patterns of presynaptic neurons are associated with those of postsynaptic neurons in the hetero-associative memory. In the storage process, 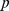 pairs of pre- and postsynaptic phase patterns, 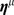 and 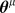 (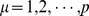), are embedded by modifying synaptic weights in accordance with an STDP learning rule. In the retrieval process, when presented with a phase pattern of presynaptic neurons which resembles the *μ*-th memory key pattern that is temporally reversed and/or stretched to 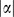 times its original timescale, 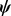 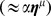 (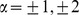), the postsynaptic neurons recall a phase pattern which resembles the associated memory output pattern that is temporally reversed and/or stretched to 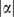 times its original timescale, 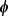 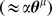.

The theoretically derived STDP window functions are compared with those reported for CA1 pyramidal neurons. The typical STDP window functions observed in the CA1 region are classified into two types [13], [14]: symmetric [15], [16] and asymmetric [17] plasticity rules. We show that both of these rules are included in a theoretically derived set of optimal STDP window functions and they allow network models with them to work as an associative memory.

## Methods

### Working Hypothesis

#### Hippocampal CA1 network works as hetero-associative memory

On the basis of the anatomical structure and physiological properties of the hippocampus, many researchers have long hypothesized that auto-associative memory resides in the CA3 region because of its recurrent connections, and hetero-associative memory resides in the CA1 region because of its feedforward connections [20]–[28].

CA1 pyramidal neurons receive inputs from the entorhinal cortex and the CA3 region, and the temporal correspondence between the activity patterns of CA1 pyramidal neurons and these presynaptic activity patterns may result in hetero-association between them by modification of the synapses onto the CA1 pyramidal neurons [27]. In line with these considerations, we assume that the hippocampal CA1 network works as hetero-associative memory and introduce a feedforward network model. The fundamental requirements for hetero-associative memory are to recall an associated activity pattern of postsynaptic neurons upon presentation of a presynaptic activity pattern.

#### Lengyel’s speculation

The STDP is an associative plasticity that adjusts synaptic efficacy depending on the relative timing of pre- and postsynaptic spikes. In the case of asymmetric STDP window functions, a synapse increases its efficacy if presynaptic spikes repetitively arrive within 5–40 msec before the postsynaptic spikes, whereas the same synapse decreases its efficacy if presynaptic spikes repetitively arrive with a similar time window after the postsynaptic spikes. On the other hand, the PRC reflects the sensitivity of oscillatory postsynaptic spike timing in response to presynaptic spike activation or a current perturbation mimicking presynaptic spike activation. The experimental protocols for measuring PRCs generate presynaptic spikes or inject perturbation currents at various timings relative to the last spike of repetitively firing postsynaptic neuron, and measure the inter-spike interval of the cycle containing the perturbation. The STDP window function and the PRC respectively indicate the effect of the timing of the presynaptic spikes relative to the postsynaptic ones on the synaptic efficacy and the timing of postsynaptic spikes, and they are based on the premise that neurons act as oscillators. In light of this, Lengyel et al. speculated that PRCs are an appropriate way to formulate neural interactions if memories are embedded by STDP [10]. In line with this speculation, we decided to search for optimal pairs of STDP window functions and PRCs.

#### Phase reduction of weakly coupled limit-cycle oscillators

The hippocampal CA1 region, as well as other regions involved in memory processing, exhibits stable oscillations of the local field potential (LFP) in several situations including awake and sleep states [29]. In such cases, the temporal order of neuronal spiking relative to the LFP oscillation are preserved [30] and correlated with the animal’s location in space [31]. This evidence suggests that memories seem to be encoded in spike times relative to ongoing LFP oscillations. Here, we formulate the storage and retrieval processes as coupled limit-cycle oscillators. As described below, under the assumption of regular firing and weak coupling, we can reduce this oscillator system to a phase equation on the basis of PRC [32], [33]. Thus, the use of PRCs is consistent with Lengyel’s speculation described above. However, the temporal spike patterns of hippocampal CA1 pyramidal neurons *in vivo* show irregular and bursting activities that differ from the regular spike activity that we assume here [29]. The phase equation derived here is valid in the limit that each neuron generates a single spike once during each period of the collective oscillations. Our assumption does not correspond to actual behaviors very well, but it is expected to capture the dominant factor of cooperative behavior in coupled oscillating systems [34]. Another big advantage of this analysis method is that it can be directly applied to real neurons by electrophysiologically measuring the PRCs [33]. Here, we use the PRCs of hippocampal CA1 pyramidal neurons recorded *in vitro* [18], [19], and predict the behavior of a virtual hetero-associative memory network composed of pyramidal neurons.

#### Retrieval of doubly spread-out patterns under the same collective theta oscillations in the running periods

It has been reported that in rats, awake neural ensemble activities are reproduced during REM episodes associated with increases in LFP theta power, and that the timescale of reactivation patterns during the REM episodes is on average twice as long as that of running periods (see Fig. 5B in [8]). This result suggests that during REM episodes, doubly spread-out patterns are reactivated under the same collective theta oscillations as those during running periods. Here, under the assumption that oscillatory dynamical properties of each neuron does not change in the CA1 network even though doubly spread-out patterns are reactivated, we use the same PRC when formulating the retrieval processes for normal and doubly spread-out patterns. This assumption simplifies the problem to find pairs of PRCs and STDP window functions optimally recalling normal and spread-out patterns.

### Minimum Model Functioning as a Temporal Hetero-associative Memory

**Figure 2:**
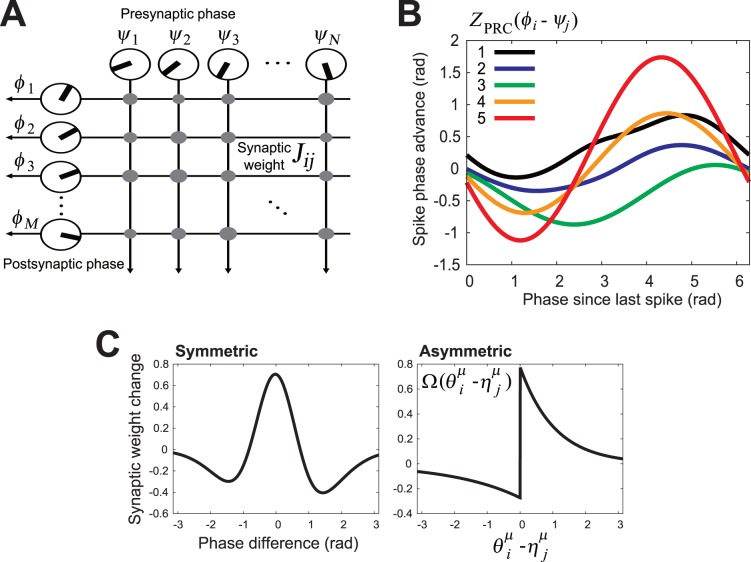
Structure of hetero-associative memory model. (A) Schematic diagram of a feedforward network with neural oscillators. Presynaptic neurons numbered 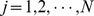 are characterized by their initial phases, 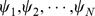, representing their individual spiking timings. The angle of the radius line in the circle represents the initial phase. Postsynaptic neurons numbered 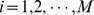 are characterized by their initial phases, 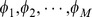. The pre- and postsynaptic neurons are fully connected by 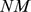 synaptic connections. (B) Phase response curves (PRCs) of hippocampal CA1 pyramidal neurons recorded *in vitro* [18], [19]. The abscissa represents the phase of a perturbation arrival, and the ordinate represents the phase shift of the postsynaptic spike in response to the perturbation current. (C) Typical STDP window functions observed in hippocampal CA1 pyramidal neurons. In the storage process, synaptic weights 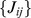 are determined in accordance with an STDP learning rule depending on the phase difference between the pre- and postsynaptic spikes. *Left*: Symmetric plasticity rule [16]. *Right*: Asymmetric plasticity rule [17].

In this study, we use the hetero-associative memory model shown in Fig. 2A. The model consists of 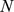 presynaptic and 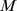 postsynaptic oscillator neurons. For simplicity, these pre- and postsynaptic neurons have the same firing period. A presynaptic neuron 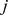 is connected to a postsynaptic neuron 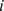 through a synapse with an efficacy (weight) 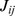. The theoretical derivations presented below assume all-to-all connectivity.

For the purpose of mathematical tractability and simplicity, we assume that the timescale of synaptic dynamics in the memory storage process is far different from that of network dynamics in the memory retrieval process. Under such an assumption of timescale separation, the storage process and retrieval process can be separated from one another.

Set out below is the outline of the hetero-associative memory functions that we wanted to study (Table 1). In the storage process, 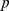 pairs of pre- and postsynaptic phase patterns, 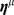 and 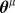 (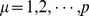), are embedded by modifying synaptic weights in accordance with an STDP learning rule. 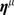 and 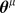 are called the 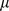-th *memory key* pattern and *memory output* pattern, respectively. In the retrieval process, after assigning a phase pattern of the presynaptic neurons that resembles the memory key pattern, 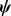 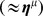, the postsynaptic neurons recall a phase pattern that resembles the memory output pattern, 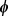 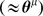. Furthermore, we treat more general cases involving the normal phase pattern retrieval described above. When presented with a phase pattern of presynaptic neurons resembling the memory key pattern that is temporally reversed and/or stretched to 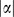 times its original timescale, 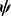 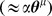 (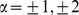), the postsynaptic neurons recall a phase pattern that resembles a memory output pattern that is temporally reversed and/or stretched to 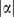 times its original timescale, 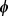 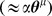. Here, 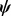 and 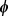 are called *retrieval key* pattern and *retrieval output* pattern, respectively. Note that the synaptic weights are then fixed during the retrieval process. The case 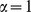 corresponds to normal phase pattern retrieval, the case 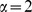 corresponds to doubly spread-out pattern retrieval, and the cases 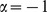 and 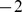 correspond to retrievals of reversed patterns. The following subsections describe the storage and retrieval processes in the network.

#### Synapse dynamics in the storage process

In the storage process, we treat 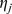 as the phase of presynaptic neuron 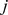 (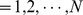), and 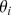 as that of postsynaptic neuron 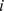 (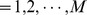). 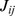 denotes the synaptic weights between presynaptic neuron 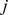 and postsynaptic neuron 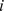. Memory storage occurs as a result of synaptic modification depending on the relative phase of the pre- and postsynaptic neurons. The amount of synaptic modification, 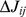, is determined according to the following synaptic plasticity rule:
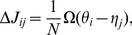
(1)where 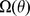 is the STDP window function. This rule is local in that the change to 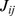 depends only on the phases of these two neurons and not on those of other neurons. When storing more than one pair (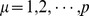), we also make a simplifying assumption that synaptic plasticity is additive across the memories:
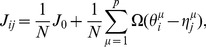
(2)where 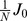 (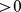) is the initial synaptic weight, which avoids negative values of 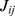. The local and additive plasticity rule is similar to the one described in the previous study [10]. The difference from the previous study is the scaling of the synaptic weight with the number of presynaptic neurons, 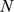. This scaling is necessary for derivation of the order parameter 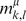, which measures the overlap between the 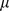-th memory key pattern 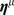 and the retrieval key pattern 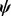, as we shall discuss later. Since the magnitude of 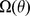 is arbitrary, there is no loss of generality due to the scaling in Eqs. (1) and (2). Each element 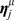 of the 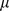-th memory key pattern 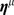 stored in the model is assigned to an independent random number in 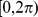 with a uniform probability, 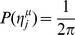. By the same token, each element 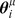 of the 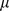-th memory output pattern 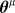 is assigned to an independent random number in 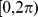 with a uniform probability, 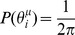. Thus, these memory patterns are not correlated with each other.

#### Network dynamics in the retrieval process

We assume that the postsynaptic neural population (Fig. 2A) consists of neural oscillators which share common features: the 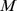 postsynaptic neurons fire rhythmically with a period 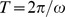 (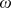 is the angular frequency), and 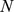 presynaptic neurons also fire with a period 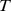. Let us consider a situation in which rhythmical firing of a postsynaptic neuron 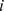 is perturbed by a total synaptic input current 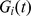 from 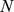 presynaptic neurons and an additive noise current 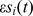:
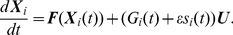
(3)


The term 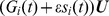 represents the driving current, in which the vector 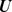 (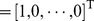) results in perturbing one degree of freedom of the neural oscillator. 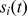 is a one-dimensional Langevin force satisfying 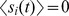, 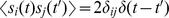, and  is the intensity of the Langevin force. 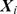 is a high-dimensional state vector that represents the activity of the postsynaptic neuron 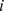, namely, the membrane potential, calcium concentration, and conductances for voltage-gated ion channels. 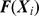 is a vector field that represents the intrinsic dynamics of neuron 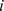. We assume that the unperturbed neural oscillator 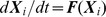 has a stable limit cycle solution:
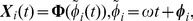
(4)where 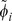 is the phase of postsynaptic neuron 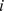, and 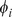 is the initial phase corresponding to the retrieval output pattern.

The total postsynaptic current 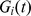 is given by
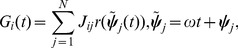
(5)where 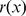 denotes the waveform of the postsynaptic current, and 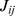 is the synaptic efficacy determining the magnitude of the synaptic current. 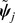 is the phase of presynaptic neuron 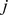, and 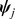 is the initial phase corresponding to the retrieval key pattern.

When 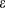 and 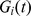 are sufficiently small, a high-dimensional system (3) can be reduced to a one-dimensional one expressing the motion of the phase 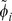 in the limit cycle orbit:
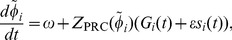
(6)where 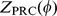 is the PRC reflecting the sensitivity to the perturbation current [32], [33]. This is called the Langevin phase equation (LPE) [35], [36].

We apply the variable transformation 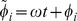 and averaging to Eq. (6). 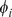 represents a slow-moving initial phase driven by a small perturbation (synaptic input) and noise. Accordingly, we can write the slow dynamics of the initial phase of the postsynaptic neuron, 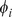, as
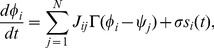
(7)

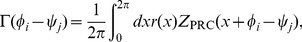
(8)




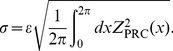
(9)



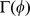
 is called the coupling function, and it is represented as a convolution of 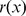 with 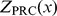. If the time constant of 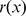 is much shorter than the period 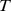 of the postsynaptic neuron, 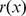 can be written as a delta function (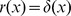). Thus, the coupling function can be written using the PRC: 
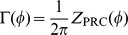
. 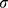 is the intensity of the noise, rescaled with the power of the PRC [36].

The effect of the white noise term 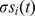 can be regarded as temporal fluctuations in the firing rate of each postsynaptic neuron around the mean 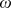 (i.e., natural frequency). Moreover, if the fluctuation strength of the measured PRC is constant with respect to the perturbation timing, the effect of 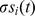 can also be regarded as a fluctuation of the measured PRC [37].

Here, we expand the STDP window function and the coupling function into their Fourier series:
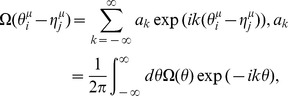
(10)

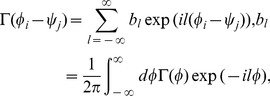
(11)where 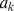 and 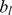 are the Fourier coefficients of the STDP window function and the coupling function (PRC), respectively. 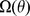 and 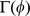, which are real-valued functions, satisfy 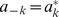 and 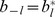 (the superscript * denotes the complex conjugate), respectively. The parameters 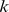 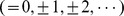 and 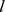 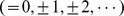 denote the wavenumbers. Note that the initial synaptic weight 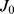 can be involved in the DC component 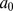 without loss of generality. Here, we define 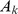 and 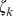 as the amplitude and the phase of 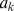, respectively (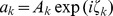). 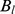 and 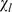 represent the amplitude and the phase of 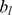 (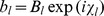). They satisfy the following equations: 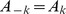, 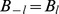, 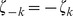, 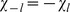.

The order parameter 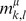, the overlap between the 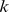-th frequency component of the 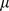-th memory key pattern 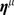 and the 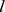-th frequency component of the retrieval key pattern 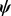, is defined as
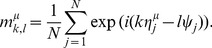
(12)


Because 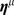 and 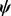 do not vary over time, each overlap 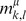 takes a constant value. Note that all the postsynaptic neurons share the same order parameter 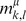 and that 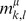 represents the characteristic function of presynaptic phase disrtibution 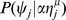 at each wavenumber: 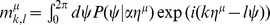.

By using 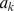, 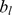, and 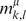, the LPE (7) can be transformed into
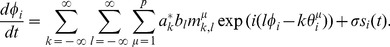
(13)


Because the postsynaptic neurons share the same 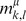 and are driven by independent noise, the 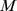 neurons can be considered to be statistically independent of each other and have the same statistical characteristics.

### Equilibrium Distribution when Storing a Finite Number of Patterns

For mathematical simplicity and tractability, we consider the case when the number of stored paired patterns 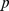 is finite in the thermodynamic limit as 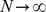 (i.e., 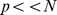). Given a retrieval key pattern similar to 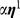, 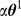 is to be retrieved. As described above, the memory key patterns 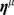 (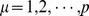) are not correlated with each other. The same goes for the memory output patterns 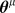.

The retrieval key pattern is generated with the following von Mises probability density function (PDF):
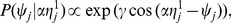
(14)where 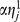 corresponds to the mean of this PDF. 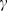 is a measure of the concentration, and it controls the similarity between the retrieval key pattern and 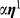.

Under the above definition of retrieval key pattern generation, each overlap 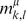 (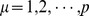) can be calculated as follows. The average overlap with 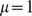 between pairs of frequency components which satisfy 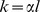 is
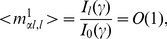
(15)where 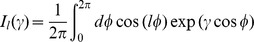 is a modified Bessel function of the first kind. On the other hand, the average overlap with 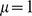 between the other components (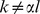) is
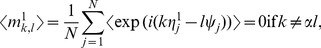
(16)and the deviation is 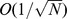. Moreover, for any 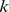 and 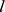, the overlap between the retrieval key 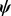 and a memory key pattern other than the first one (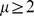) is on average
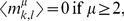
(17)and the deviation is 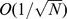. In the case of a finite 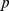, the number of terms with 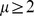 in Eq. (13) is finite. Moreover, the Fourier coefficients 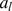 and 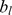 rapidly approach zero as 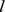 increases. Thus, contributions of the terms except those with 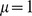 and 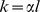 to Eq. (13) can be neglected in the limit 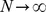. The phase dynamics can be rewritten as



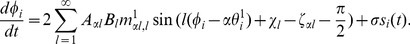
(18)Note that the term consisting of DC components 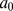 and 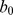 in Eq. (13) is a constant. We can safely neglect this term, because the constant term can be involved in the natural frequency 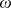 without loss of generality. Equation (18) shows that the statistical properties of the hetero-associative memory model in the case of finite loading (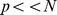) and those in the simplest case of just one pair of patterns to be stored (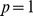) are identical.

From Eq. (18), we obtain the equilibrium phase distribution of each postsynaptic neuron:
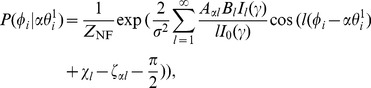
(19)

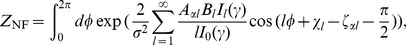
(20)where 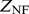 is the normalizing factor.

### Mutual Information per Neuron

Mutual information, which measures how related two random variables are, is nonnegative and takes 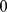 only if these variables are independent.

When recalling the 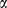 times spread-out memory pattern 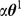, the mutual information of the retrieval output 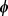 relative to 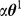 per neuron, 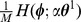, is given by
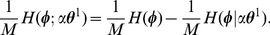
(21)


Here, 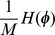 is the entropy of 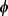 per neuron, which measures the uncertainty associated with 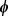. 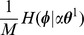 is the conditional entropy of 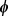 given 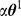, which quantifies the remaining uncertainty of 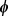 given that 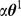 is known. Because each postsynaptic neuron is statistically independent of each other and has the same statistical characteristics as described above, 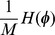 and 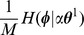 can be simply written as follows:
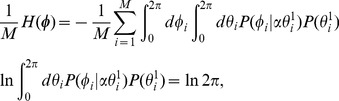
(22)

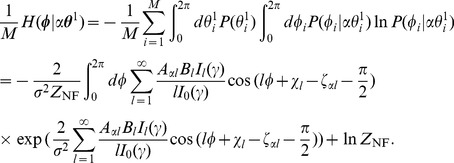
(23)


Because 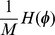 is a constant, as shown by Eq. (22), maximization of the mutual information 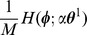 in Eq. (21) is identical to minimization of the conditional entropy 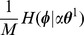 in Eq. (23).

Note that in Eq. (21), for any 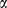, the value of 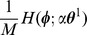 is equal to that of the reverse pattern retrieval case, i.e., 
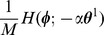
. This is because the system is symmetric with respect to sign inversion of the key and output patterns. Namely, the system, which can retrieve normal and doubly spread-out patterns, can also retrieve reversed patterns with the same quality.

In what follows, we search for pairs of PRCs and STDP window functions that are optimal for retrieving both normal and doubly spread-out patterns by jointly maximizing two object functions, 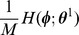 and 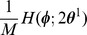. To solve this joint optimization problem, we employ a simple sum of these functions,
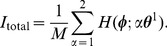
(24)


Furthermore, for comparison, we also use an objective function,
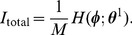
(25)


As mentioned above, the optimal system derived by maximization of the objective function is also optimal for retrieving reversed patterns.

### Phase Response Curve of Hippocampal CA1 Pyramidal Neurons

In our previous work, we obtained PRCs from rat hippocampal CA1 pyramidal neurons by performing whole-cell patch-clamp recording *in vitro* [18], [19]. Figure 2B shows the PRCs from that research. In the protocol for measuring PRCs, we injected DC depolarizing currents into the somata of rat CA1 pyramidal neurons to evoke periodic firing. Using the dynamic clamp, the mean inter-spike interval (ISI) was adjusted to the target period by tuning the DC depolarizing current. In measuring these PRCs, the firing frequencies of those neurons were tuned in the theta frequency range (4–14 Hz). Next, we evoked a one-shot rectangle perturbation superimposed on the DC depolarizing current by using various timings relative to the spike, and measured how the perturbation current disturbed the timing of the succeeding spike, i.e., phase response. The spike times randomly fluctuated due to intrinsic noise in the neurons. To extract the PRCs from the noisy data of the phase responses, we made use of a PRC measurement model formulated as an LPE [38] (the same as the one used in the current study) and applied a maximum a posteriori (MAP) estimation algorithm based on the measurement model to the noisy phase response data. The effectiveness of the measurement model and the reliability of the estimated PRCs were verified by demonstrating that the LPEs with the estimated PRCs could predict the stochastic behaviors of the same neurons, whose PRCs had been measured, when they were perturbed by various periodic stimulus currents [18]. A detailed explanation of the experimental conditions and the MAP estimation algorithm can be found in [36], [38], [39], while the reliability and quality of PRCs used here has been discussed in detail in [18], [19].

## Results

### Performance of Hetero-associative Memory Model with Typical Parameters

We carried out numerical simulations on the hetero-associative memory model with typical parameters, and we compared the numerical results with theoretical predictions. Here, we use the hetero-associative memory model endowed with a PRC (cell 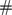
1 in Fig. 2B [18]) and a typical STDP window function (*left* panel of Fig. 2C [16]) of CA1 pyramidal neurons. In the following numerical simulations, we embedded three pairs of random phase patterns, 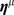 and 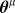 (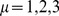) in the synaptic weight 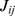. We used the following to evaluate the retrieval quality of this model:
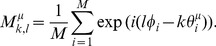
(26)


This measure is the overlap between the 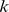-th frequency component of the 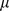-th memory output pattern 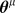 and the 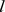-th frequency component of the retrieval output pattern 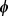. At equilibrium, the overlap 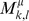 can be theoretically obtained with the PDF (19):
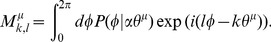
(27)


As shown in Eq. (27), 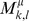 represents the characteristic function of the postsynaptic phase distribution 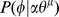 at each wavenumber.

**Figure 3:**
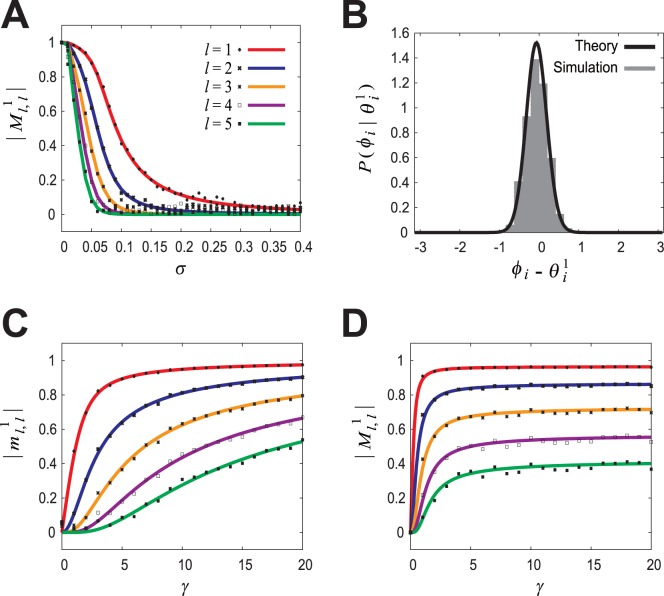
Performance of hetero-associative memory model with typical parameters. We use a typical STDP window function (*left* panel of Fig. 2C [16]) and the PRC (cell 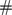
1 in Fig. 2B [18]) measured from hippocampal CA1 pyramidal neurons. In this simulation, 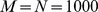. Given a retrieval key pattern similar to 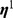, 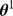 is to be retrieved (i.e., normal spike pattern retrieval). (A) Amplitudes of the overlaps 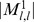 (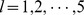 denotes the wavenumber) at equilibrium as a function of the noise intensity 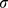 when 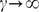. As defined in Eq. (26), 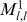 is the overlap between the first memory output pattern 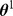 and the retrieval output pattern 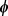 in the 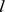-th frequency component: 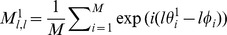. 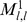 represents the characteristic function of the postsynaptic phase distribution 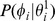 at each wavenumber 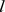. Solid curves are theoretical results obtained from Eq. (27); The plotted points are from numerical simulations using LPE (13). (B) An example of the PDF (19) and a histogram of phase difference 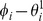 obtained by numerically solving the LPE (13) at equilibrium. 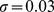 and 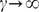. (C) Amplitudes of the overlaps 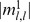 (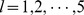) as a function of the concentration parameter 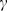. As defined in Eq. (12), 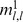 is the overlap between the first memory key pattern 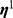 and the retrieval key pattern 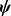 in the 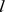-th frequency component: 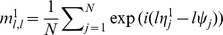. 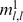 represents the characteristic function of the presynaptic phase distribution 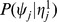 at each wavenumber 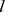. Solid curves are theoretical results obtained from Eq. (15); Plots are obtained from a retrieval key pattern randomly generated with the von Mises PDF (14). (D) Amplitudes of the overlaps 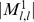 (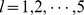) at equilibrium as a function of 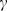. 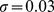. Solid curves are theoretical results obtained from Eq. (27); The plots are from numerical simulations using LPE (13).

First, we verified the effect of intrinsic noise on the retrieval quality of this model. For simplicity, we gave it a retrieval key pattern identical to the normal memory key pattern (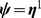), which corresponds to the special case of 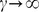. Figure 3A plots the amplitude of the overlap 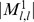 (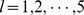) at equilibrium as a function of the noise intensity 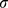. In this figure, the LPE (13) was solved numerically by using the Euler method, and the values of 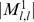 calculated with Eq. (26) were compared with theoretical predictions obtained by Eq. (27). As shown in Fig. 3A, the numerical results coincide with the theoretical values for all wavenumbers 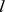. When the noise intensity is sufficiently small (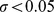), the retrieval output pattern 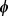 has an appreciable overlap with 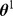, i.e., 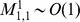. As 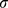 increases, the overlap 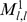 approaches zero. Figure 3B shows an example of the PDF (19) and a histogram of the phase difference 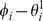 obtained by numerically solving LPE (13) at equilibrium. Here, 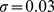 and 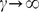. In this figure, the PDF (19), which forms a unimodal distribution, is in good agreement with the histogram normalized by the bin width.

Next, we verified the effect of the degraded retrieval key patterns on the retrieval quality of this model. We generated the phase patterns by using the conditional PDF (14) given 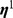 and various values of 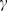, and we used these generated patterns as retrieval key patterns. Figures 3C and D respectively show amplitudes of the overlaps 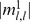 (defined in Eq. (12)) and 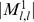 (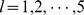) at equilibrium as a function of 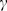. Here, 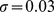. The numerical results coincide with the theoretical ones for all 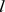. When 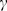 is sufficiently large (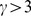), the retrieval output pattern 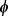 has an appreciable overlap with 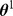, i.e., 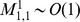. As 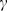 decreases, the overlap 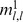 approaches zero faster than 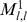 converges to zero.

Note that we got similar results to those above by using the other PRCs (different from cell #1 in Fig. 2B) and another STDP window function (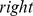 panel of Fig. 2C).

### STDP Window Functions Optimally Matched to PRCs of Hippocampal CA1 Pyramidal Neurons

We searched for STDP window functions optimally matched to the PRCs of the five hippocampal CA1 pyramidal neurons shown in Fig. 2B. As described in the Methods section, we considered the two cases. One is that we maximize the objective function 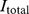 defined in Eq. (25) to search for STDP window functions that are optimal for retrieving normal patterns. The other is that we maximize the objective function 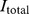 defined in Eq. (24) to search for STDP window functions that are optimal for retrieving normal and doubly spread-out patterns. In both cases, we assigned the Fourier coefficients of the PRCs of the hippocampal CA1 pyramidal neurons to 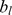 of each mutual information constituting the objective function 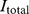, and under the constraint of the measured PRC, searched for 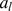, the Fourier coefficients of the STDP window functions to maximize the objective function 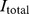. Note that a system which can optimally retrieve normal and doubly spread-out patterns can also retrieve reversed ones, because it is symmetric with respect to sign inversion of the key and output patterns.

Because the mutual information 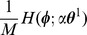 in Eq. (21) monotonically increases as 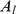 (the amplitude of 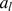) increases, we imposed the following constraint condition on the power of STDP window function:
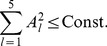
(28)


Here, we truncated the Fourier series of the PRCs and STDP window functions after the fifth term. Referring to the value of the STDP power in a previous study [10], we set 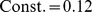. To solve this optimization problem, we used the FMINCON function in the Matlab Optimization Toolbox.

**Figure 4:**
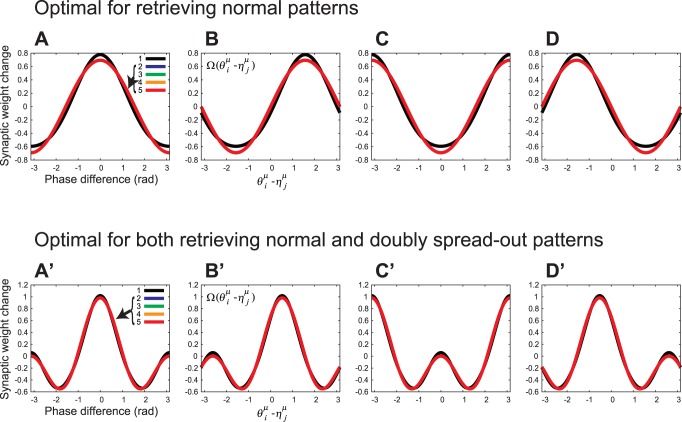
Examples of STDP window functions optimally matched to PRCs of five hippocampal CA1 pyramidal neurons shown in Fig. 2B. (A–D) By maximizing the objective function 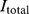 defined in Eq. (25), we searched for STDP window functions that are optimal for retrieving normal patterns. (A′–D′) By maximizing the objective function 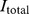 defined in Eq. (24), we searched for ones that are optimal for both retrieving normal and doubly spread-out patterns. In all cases, 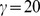, 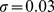. We obtained connected sets of optimal STDP window functions, as described in the main article. Each of the four panels in the upper and lower rows plots examples of optimal STDP window functions with different phases. The numbers assigned to each line correspond to the cell indexes in Fig. 2B. All sets of optimal STDP window functions except for cell #1 have the same form. (A, A′) STDP window functions when 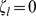, which corresponds to the symmetric STDP rule. (B, B′) STDP window functions when 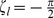 (B) and 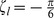 (B′), which correspond to the asymmetric STDP rule. (C, C′) STDP window functions when 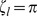, which corresponds to the inverted symmetric STDP rule. (D, D′) STDP window functions when 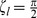 (D) and 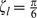 (D′), which correspond to the inverted asymmetric STDP rule.

First, we searched for STDP window functions that are optimal for retrieving normal patterns. By maximizing the objective function in Eq. (25) under the power constraint (28), we obtained a connected set of optimal STDP window functions described as 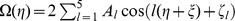 for any 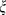 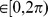, in which all members have the same shape, because if 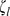 and 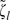 (the phases of 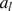) satisfy 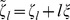 for all 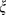 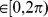, the values of 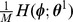 are exactly the same in both cases. Figures 4A–D show five sets of optimal STDP window functions; each set is optimally matched to each PRC of the five different CA1 pyramidal neurons shown in Fig. 2B. The four panels of Figs. 4A–D plot examples of optimal STDP window functions with different phases 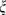. All sets of functions except for cell #1 have the same form, and they are almost composed of fundamental frequency components. The shape of the optimal STDP window function obtained from the PRC of cell 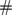
1 is very similar to the others, even though the PRC of cell #1 contains a relatively large number of higher frequency components compared with the PRCs of the other cells (see Fig. 2B).

Next, we searched for STDP window functions that are optimal for both retrieving normal and doubly spread-out patterns. By maximizing the objective function in Eq. (24) under the power constraint (28), we obtained a connected set of optimal STDP window functions in the same manner as described above. Figures 4A′–D′ show five sets of optimal STDP window functions; each set is optimally matched to each PRC of the five different CA1 pyramidal neurons shown in Fig. 2B. The four panels of Figs. 4A′–D′ plot examples of optimal STDP window functions with different phases 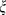. All sets of functions except for cell #1 have the same form, and they are almost completely composed of fundamental and second frequency components. The shape of the optimal STDP window function obtained from the PRC of cell #1 is very similar to the others, even though the PRC of cell #1 contains a relatively large number of higher frequency components compared with the PRCs of the other cells (see Fig. 2B).

The results of Fig. 4 suggests that the optimal STDP window function depends heavily on the fundamental frequency components of the PRCs, and thus its shape is nearly invariant for all of the PRCs of the CA1 pyramidal neurons. In addition, the joint optimization for retrieving normal and doubly spread-out patterns only yielded the second frequency components of the STDP window functions, and thus, the second frequency components of the STDP window function play a key role in recalling doubly spread-out phase patterns.

**Figure 5:**
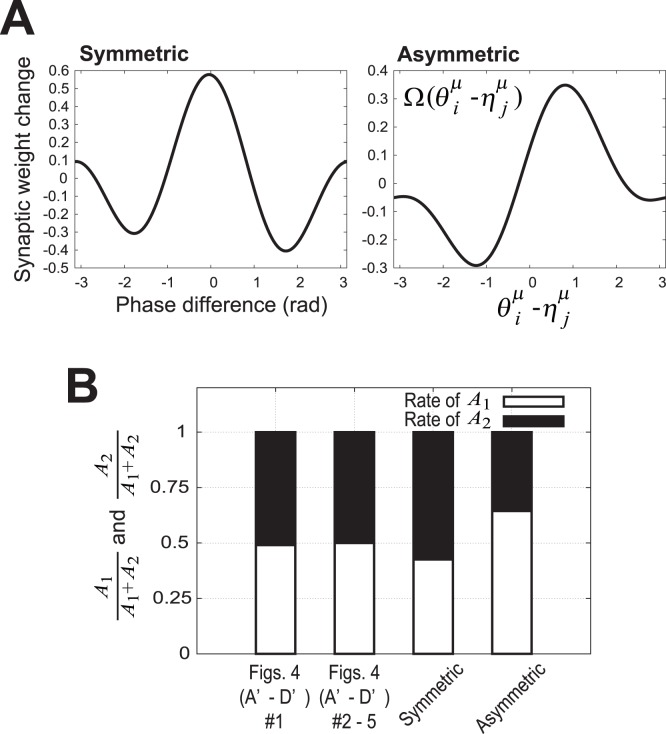
Comparison of purposely designed STDP window functions (Figs. 4A′–D′) and those reported for the hippocampal CA1 region. We computed the Fourier series of symmetric and asymmetric STDP window functions in Fig. 2C and compared the first two frequency components of the STDP window functions in Fig. 2C with those in Figs. 4A′–D′. (A) Symmetric and asymmetric STDP window functions composed of only the fundamental and second frequency components of the ones in Fig. 2C. *Left*: Symmetric plasticity rule [16]. *Right*: Asymmetric plasticity rule [17]. (B) Rates of fundamental and second frequency components of STDP window functions in Fig. 5A and the purposely designed ones in Figs. 4A′–D′. We compared the amplitudes between the two Fourier coefficients of each STDP window function, i.e., 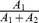 and 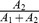. Symmetric: *left* panel of Fig. 5A [16]. Asymmetric: *right* panel of Fig. 5A [17].

It has been reported that there are two types of STDP window functions in hippocampal CA1 pyramidal neurons [13], [14], i.e., symmetric (*left* panel of Fig. 2C [16]) and asymmetric (*right* panel of Fig. 2C [17]). Here, we compared physiologically measured window functions with purposely designed ones for memory recalls. We computed the Fourier series of symmetric and asymmetric STDP window functions in Fig. 2C and compared the fundamental and second frequency components of the STDP window functions in Fig. 2C with the frequency components of the ones in Figs. 4A′–D′. Figure 5A plots symmetric and asymmetric STDP window functions composed of only the fundamental and second frequency components of the ones in Fig. 2C. The purposely designed STDP window functions shown in Figs. 4A′ and B′ qualitatively conform to those of Fig. 5A. Figure 5B shows the rate of the fundamental and second frequency components for STDP window functions in Fig. 5A and the purposely designed ones in Figs. 4A′–D′. We compared the amplitudes between the two Fourier coefficients of each STDP window function: 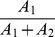 and 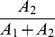. As shown in Fig. 5B, the joint optimization for retrieving normal and doubly spread-out patterns yields equal amounts of fundamental frequency component and second frequency component (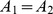), and the amount of second frequency component in the symmetric and asymmetric STDP window functions in Fig. 2C is almost equal to that of the fundamental frequency components.

Moreover, Figs. 4C′ and D′ show an inverted symmetric window function and inverted asymmetric one in contradistinction to Figs. 4A′ and B′. These window functions were found in regions outside the hippocampal CA1 area (see [40]–[42]).

### Memory Retrieval in the Hetero-associative Memory Model

By using numerical simulations, we confirmed that the system with the STDP window functions in Figs. 4A′–D′ can function as intended. The synaptic weight 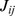 was determined using the STDP window function (cell #5 in Fig. 4A′) to store three pairs of random phase patterns, 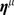 and 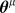 (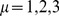), and the retrieval performance of the system with the determined synaptic weight and the measured PRC (cell #5 in Fig. 2B) was verified. In the following simulations, we used the retrieval key pattern generated with the conditional PDF (Eq. (14)) given 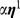, and under this condition, we checked whether the system could recall a temporally reversed memory output pattern and/or one stretched to 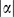 times its original timescale, 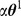. The overlap 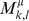 between the 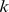-th frequency component of 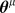 and the 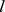-th frequency component of 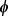 (defined in Eq. (26)) was used as a measure of retrieval performance.

**Figure 6:**
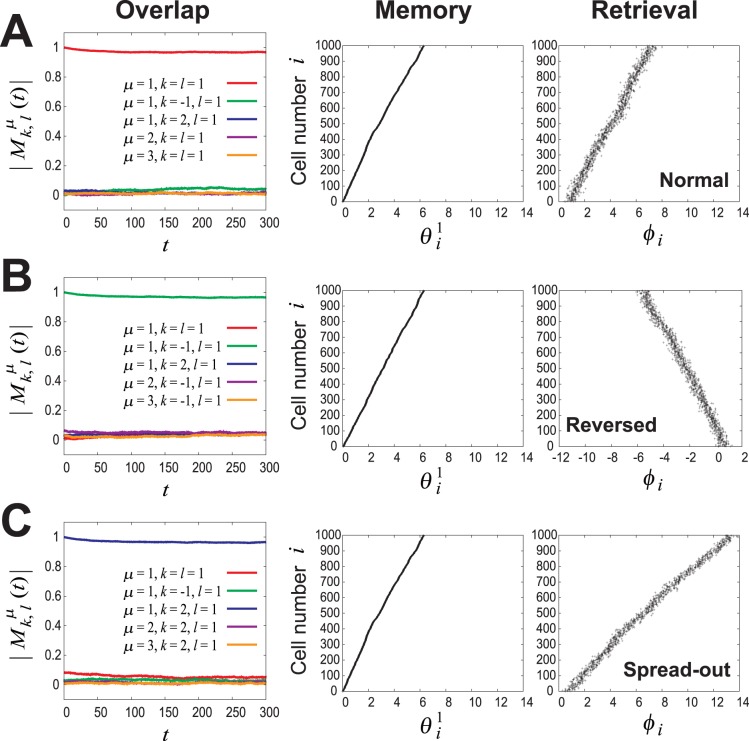
Confirmation that the system with the STDP window functions in Figs. 4(A′–D′) can function as intended. The synaptic weight 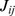 was determined using the STDP window function (cell 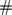
5 in Fig. 4A′) to store three pairs of random phase patterns, 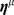 and 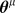 (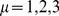), and when presented with the retrieval key pattern generated with the conditional PDF (Eq. (14)) given 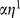, the retrieval performance of the system with the determined synaptic weight and the measured PRC (cell #5 in Fig. 2B) was verified by using numerical simulations (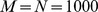, 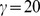, 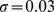). (A) Normal spike pattern retrieval (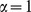). (B) Reversed pattern retrieval (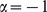). (C) Doubly spread-out pattern retrieval (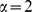). *Left column*: Time evolution of the amplitude of the overlap between the 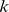-th frequency component of 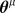 and the 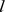-th frequency component of 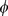, 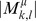. *Center column*: An example of the memory output pattern as originally stored, 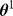. *Right column*: The retrieval output pattern 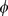 at equilibrium (corresponding to 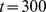 in *left column*).

The three panels of the *left column* in Fig. 6 show the time evolution of 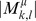 in the cases of the normal, reversed, and spread-out pattern retrievals. 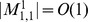 and the others are almost zero in the normal pattern retrieval, whereas 
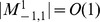
 and the others are almost zero in the reversed pattern retrieval, and 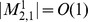 and the others are almost zero in the doubly spread-out pattern retrieval. The panels of the *center* and *right columns* in Fig. 6 show samples of memory output patterns and retrieval output patterns at equilibrium in the cases of the normal, reversed, and doubly spread-out pattern retrievals. We also confirmed that the system with the other STDP window functions in Figs. 4B′–D′ and the other PRCs (different from cell #5 in Fig. 2B) works just as well as these.

## Discussion

### Summary of Results and Conclusions

By maximizing the objective functions given by the mutual information, we derived pairs of STDP window functions and PRCs optimally recalling normal, reversed, and doubly spread-out phase patterns in a hetero-associative memory model. We searched for a set of optimal STDP window functions using the measured PRCs from *in vitro* experiments on hippocampal CA1 pyramidal neurons.

The optimal STDP window function heavily depends on the fundamental frequency component of the PRCs, and thus its shape is almost invariant with respect to the PRCs of CA1 pyramidal neurons (see Fig. 4). Even though the PRC of cell #1 contains a relatively large number of higher frequency components compared with the PRCs of the other cells (see Fig. 2B), the shape of the optimal STDP window function obtained from the PRC of cell #1 is very similar to the others. A comparison of results in Figs. 4A–D and A′–D′ suggests that second frequency components of STDP window functions play a key role in recalling doubly spread-out phase patterns. If the memory key and output patterns are respectively assigned to an independent number in 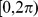 with a uniform probability, in the limit of 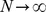, the doubly spread-out patterns are orthogonal to the original patterns (i.e., 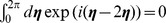). Because of this orthogonality, this system can retrieve spread-out patterns if the STDP window function contains higher frequency components.

As shown in Fig. 6B, the system, which can retrieve normal and doubly spread-out phase patterns, can also retrieve reversed patterns. This is because the symmetry with respect to sign inversion for key and output patterns is satisfied as it is in the conventional associative memory model [43], [44]. Thus, the mutual information in retrieving normal and doubly spread-out phase patterns is equal to the information involved in retrieving the reversed patterns.

Furthermore, the mutual information is invariant with respect to the phases of the STDP window functions and PRCs. Thus, the set of optimal STDP window functions forms a connected set homeomorphic to a ring, examples of which have a good qualitative match to those reported in hippocampal CA1 pyramidal neurons, such as the symmetric [15], [16] and asymmetric window functions [17]. Note that the original data from Wittenberg and Wang (2006) exhibit a phase delay between the pre- and postsynaptic spikes in the peak of the symmetric STDP window function [16]. On the other hand, we used a simplified STDP window function (*left* panel of Fig. 2C) that ignored the phase delay in the peak. This is because the phase shift of the STDP window function has no effect on the retrieval performance of the associative memory model, as stated above. As shown in Fig. 5B, the fundamental and second frequency components of STDP window functions reported for CA1 neurons have roughly the same scale, which coincides with those of the theoretically derived STDP window functions.

Thus, the results obtained here suggest that the properties of the neural interaction and the synaptic plasticity rule in the CA1 region support a hetero-associative memory function recalling normal, doubly spread-out, and reversed patterns.

### Effect of STDP Multiplicity in Single Neurons in Recalling Memories

Optical imaging studies have suggested that the shape of STDP window function in the CA1 pyramidal neuron depends on the location on the dendrite [13], [14]. A symmetric STDP window function was observed in the proximal-to-soma dendrite, whereas an asymmetric STDP window function was observed in the distal-to-soma dendrite.

Here, we verify the effects of symmetric and asymmetric STDP window functions in single neurons on recall memory. We assume that synapses between a postsynaptic neuron 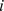 (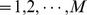) and a presynaptic neuron 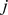 (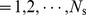) obey the symmetric STDP rules, and others between a postsynaptic neuron 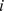 (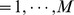) and a presynaptic neuron 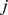 (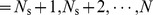) obey the asymmetric STDP rules. 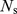 is the number of synapses obeying the symmetric STDP rules in a single neuron. For mathematical simplicity, the symmetric and asymmetric STDP window functions coexisting in single neurons are described as
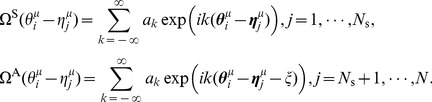
(29)


Here, the variable 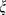 is the phase difference between the symmetric and asymmetric STDP window functions. The typical value of 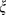 is 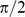. Under this condition, we can rewrite Eq. (13) as follows:
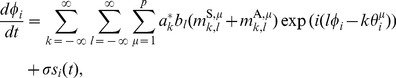
(30)

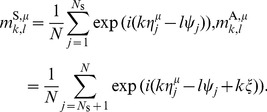
(31)


Furthermore, in the limit of 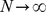 and 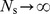, while keeping the 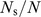 ratio constant at 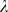, the sum of the partial overlaps, 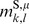 and 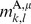 becomes
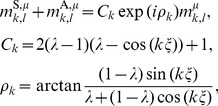
(32)where 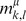 is the same as the order parameter defined in Eq. (12). Thus, the above model is essentially equivalent to the homogeneous STDP model (Eq. (13)) except for the existence of the coefficient 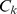 and the phase 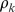. If the fundamental frequency components are dominant in the PRCs, this inhomogeneous model can also work as a hetero-associative memory. Figure S1 (supporting information) shows the results of numerical simulations when 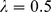. Because 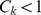 if 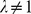 or 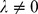, the retrieval quality becomes worse than that of the homogeneous model.

### Difference between Our Approach and Lengyel’s

Lengyel et al. (2005) tried to determine whether the properties of neural interactions and the synaptic plasticity rule in the CA3 region support an auto-associative memory function [10]. Figure S2 (supporting information) illustrates the top-down approach they used. First, they developed a theory treating auto-associative memory retrieval as a kind of Bayesian inference and constructed a gradient ascent algorithm for the MAP estimation. Next, they reinterpreted this algorithm as phase oscillators consisting of PRCs and STDP window functions. Finally, they qualitatively compared the PRCs of hippocampal CA3 pyramidal neurons with ones theoretically derived from a typical STDP window function. As a result of their contrasting the top-down approach with Marr’s tri-level hypothesis [45], their study can be considered as a bridge from the computational level to the algorithmic level. Furthermore, they tried to bridge the algorithmic and physical levels by reinterpreting the algorithm they derived as phase oscillators.

On the other hand, contrasting our approach shown in Fig. 1 with Marr’s tri-level hypothesis, our study can be considered as a bridge between the physical level and the algorithmic level. Note that the phase equation (e.g., Eq. (13)) reduced from the coupled oscillator system (Eq. (3)) corresponds to the algorithm of hetero-associative memory. Unfortunately, there is no clear relationship between our phase equation derived from the bottom up and Lengyel’s gradient ascent algorithm derived from the top down at this moment. In the future, we will explore the correspondence between our results and Lengyel’s. [10].

### How and Where are Key Patterns for Recalling Reversed and Spread-out Patterns Created?

As shown in Fig. 2, a key pattern has to be input in order to recall its associated output pattern in a hetero-associative memory. As summarized in Table 1, to retrieve reversed and spread-out patterns, the associated key patterns also have to be reversed and spread-out. Thus, we must answer new questions as to where and how key patterns for recalling reversed and spread-out patterns are created. It is possible for a recurrent network such as the CA3 network to create and provide reversed and spread-out patterns. The CA3 region provides one of the dominant inputs to the CA1 region [25], [27]. By applying mean field approximations, our theory of hetero-associative memory can be straightforwardly extended to analyses of auto-associative memory. The preliminary results suggest that the properties of sine-like PRCs of hippocampal CA3 pyramidal neurons recorded by Lengyel et al. (2005) [10] and the typical STDP rule can support an auto-associative memory function for recalling reversed and spread-out phase patterns. Thus, the preliminary results and the results of this paper indicate the possibility that a combination of the CA1 network and the CA3 network can consistently work to retrieve reversed and spread-out patterns. We will report on this issue in our next study.

### Role of Reversed and Spread-out Pattern Retrievals

It has been considered that memories are first stored in the hippocampus and are gradually moved to the neocortex in a more permanent form of storage. Temporally spread-out pattern retrieval, in which the temporal order of the memory spike sequence is preserved and the timescale of retrieval pattern is about two times longer, may be important for the memory translation and system consolidation [8]. On the other hand, temporally reversed pattern retrieval is suggestive of evaluating event sequences in the manner of reinforcement learning models [46]. During waking periods, reversed pattern retrieval occurs *in situ*, allowing immediately preceding events to be evaluated in precise temporal relation to the current one, and so it may be an integral mechanism for learning about recent events [9].

## Supporting Information

Figure S1
**Effect of coexisting symmetric and asymmetric STDP window functions in single neurons on the memory retrieval.** In this numerical simulation, we used the PRC (cell #2 in Fig. 2B) and symmetric and asymmetric STDP window functions (cell #2 in Figs. 4A′ and 4B′) at the same rate in single neurons. 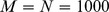, 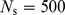, 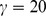, 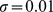. (A) Normal spike pattern retrieval (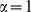). (B) Reversed pattern retrieval (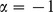). (C) Doubly spread-out pattern retrieval (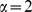). *Left column*: Time evolution of the amplitude of the overlap between the 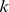-th frequency component of 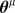 (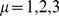) and the 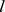-th frequency component of 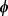, 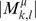. *Center column*: An example of the memory output pattern as originally stored, 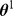. *Right column*: The retrieval output pattern 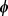 at equilibrium (corresponding to 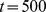 in *left column*).(EPS)

Figure S2 **Outline of the previous study by Lengyel et al. (2005)** [**10**] **.** They derive pairs of PRCs and STDP window functions for optimally recalling the originally stored phase pattern.
(EPS)
